# Reactive Compatibilization of PLA/PA11 Blends and Their Application in Additive Manufacturing

**DOI:** 10.3390/ma12030485

**Published:** 2019-02-05

**Authors:** Damien Rasselet, Anne-Sophie Caro-Bretelle, Aurélie Taguet, José-Marie Lopez-Cuesta

**Affiliations:** IMT Mines Ales, C2MA, 6 avenue de Clavières, 30319 Alès CEDEX, France; damien.rasselet@mines-ales.fr (D.R.); anne-sophie.caro@mines-ales.fr (A.-S.C.-B.); aurelie.taguet@mines-ales.fr (A.T.)

**Keywords:** PLA, PA11, polymer blends, compatibilization, additive manufacturing

## Abstract

The aim of this work was to study the properties of polylatic acid/polyamide 11 (PLA/PA11) blends compatibilized with a multifunctionalized epoxide, Joncryl^®^, and to evaluate the performance of such blends processed by Fused Deposition Modeling (FDM) 3D printing, compared to those produced by injection molding method. Blends containing different Joncryl contents from 0.5 to 3 wt% were prepared by twin-screw extrusion. Evaluation of thermal, rheological and mechanical properties of such blends proved that Joncryl acted as a compatibilizer. Results showed that Joncryl effects on blends properties were improved with increasing its content. A significant reduction of PA11 dispersed phases diameter and an improvement of tensile properties with a ductile behavior were achieved for the highest Joncryl contents. A significant elongation of PA11 dispersed phases was observed into FDM filaments and dog bone shaped specimens produced thereafter. Despite this peculiar morphology, FDM printed samples exhibited only enhanced stiffness but poor reinforcement and elongation at break in comparison with injected ones.

## 1. Introduction

Polylactic acid (PLA) is one of the bio-based polymers that generate the most interest, because of its biodegradability, high tensile strength and modulus. Nevertheless, its relatively poor thermal stability and significant brittleness may be major drawbacks for other large-scale commercial applications than packaging. To overcome these drawbacks, the most used and studied methods consist of adding nanoscaled mineral fillers (nanoparticles) to get a PLA-based nanocomposite or blending PLA with other polymers, bio-based as well as oil-based [1,2,3,4,5,6,7,8,9]. The latter strategy has been widely studied for PLA, because polymer blending is an efficient method commonly used at industrial scale, allowing properties of different polymers to be combined in order to obtain a new material with desirable properties. To achieve this objective, compatibilization is often required to improve polymer compatibility and control morphology.

In this context, polylactic acid/polyamide 11 (PLA/PA11) blends represent a good solution to get materials with improved thermomechanical properties compared to the ones of neat PLA. Indeed, polyamide 11 (PA11) is a bio-based polymer derived from castor oil with excellent thermal stability and high elongation at break and impact strength. Moreover, interfacial interactions and reactions, better known as interchange reactions, could potentially occur between polyamides and polyesters during melt blending, leading to an improvement of compatibility [10,11,12,13,14,15]. Hence, PA11 has been recently considered as a good candidate for blending with PLA. 

Even if some studies have concluded to a partial compatibility with high interfacial interactions between PLA and PA11 [16,17,18], literature shows that the immiscibility and incompatibility of these polymers prevail, which makes compatibilization necessary. Various strategies were studied, most of them dealing with reactive pathways by adding catalyst [17,19] to promote ester-amide interchange reactions, or reactive copolymer containing glycidyl methacrylate (GMA). Such copolymers are interesting, because GMA is a chemical moiety with epoxide functions, which can react with both hydroxyl and carboxyl reactive functional end chains of PLA and amine and carboxyl end groups of PA11. This coupling reaction at interface improves PLA/PA11 adhesion and compatibilizes the blend.

Dong et al. [20] have shown that ethylene glycidyl methacrylate-graft-styrene-co-acrylonitrile rubber (EGMA-g-AS) has no compatibilizing role in PLA/PA11 blends. Whatever the mixing sequence tested, EGMA-g-AS was never located at the interface and no decrease of the dispersed PA11 domains size was observed. Despite this, the development of a salami structure and the localization of EGMA-g-AS in PLA matrix led to better mechanical properties, with a large increase of ductility and impact strength, compared to the ones of neat blend. 

Walha et al. [21] investigated the effects of incorporation of Joncryl^®^ on the rheological, morphological and mechanical properties of PLA/PA11 blends. Joncryl^®^ is a styrene-acrylic multifunctional epoxy copolymer, usually employed as chain extender to enhance polyesters thermal stability. Two main methods of mixing were used: the first one consisted of introducing all compounds simultaneously in the extruder and the second one consisted of modifying PLA by premixing it with Joncryl and then adding PA11. Authors demonstrated the role of Joncryl as a compatibilizer for the PLA/PA11 system, by the significant decrease of the size of PA11 dispersed phases and the interfacial tension as well as the improvement of ductility, especially with the second mixing route. Similar results were obtained by Zolali et al. [22], that used ethylene methyl acrylate-glycidyl methacrylate to compatibilize a co-continuous PLA/PA11 blend. 

The aim of this work was to study the properties of PLA/PA11 blends compatibilized with a multifunctionalized epoxide, Joncryl^®^. Compared to Walha et al., our works were dedicated to optimize mechanical properties of a PLA/PA11 80/20 wt% blend with an extended range of Joncryl percentage, and to determine the effects of Joncryl on blends morphology and properties. 

The second objective was to evaluate the performance of such blends processed by Fused Deposition Modeling (FDM) 3D printing, compared to those produced by injection molding method. To the best of our knowledge, this is the first study addressing the application of PLA/PA11 blend for 3D printing, especially FDM. Additive manufacturing or 3D printing recently gained an increasing interest in industry as well as academic area. Additive manufacturing gathers a lot of processing techniques, having the ability to produce complex geometries pieces layer by layer with low cost production, and exhibiting numerous advantages associated with rapid prototyping. But up to now, few compatible materials with 3D printing are available on the market, restricting its tremendous potential. PLA is one of the most used polymers for this technology, and PLA based blends are a good solution to develop materials with specific required properties and to expand the range of 3D printable materials [23,24,25,26,27,28].

In the first part, the morphology of blends with different Joncryl contents will be examined to assess the effectiveness of Joncryl as compatibilizing agent. Then, the effects of Joncryl on thermal and rheological properties will be evaluated. In the second part, a study of the influence of FDM process on the morphology and mechanical properties obtained for compatibilized blends, compared to the ones obtained for injected samples, will be performed.

## 2. Materials and Methods 

### 2.1. Materials

**Figure 1:**
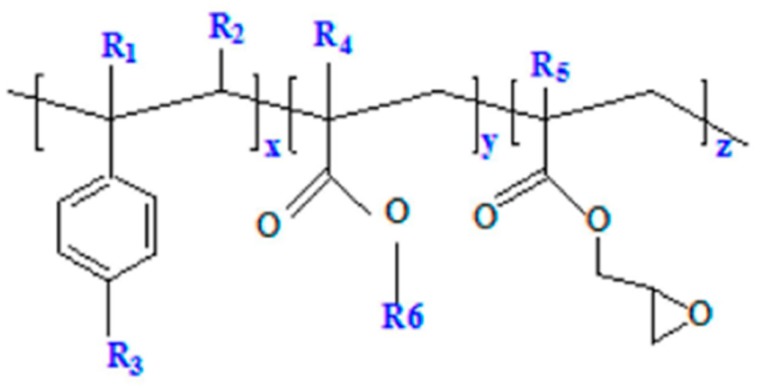
Chemical structure of Joncryl ADR^®^-4368, and the general structure of the styrene-acrylic multifunctional oligomeric chain extenders. R_1_–R_5_ are H, CH_3_, a higher alkyl group or combinations of them; R_6_ is an alkyl group. x, y and z are between 1 and 20.

The polylactide (PLA grade 3251D) used in this study was purchased from NatureWorks (Minnetonka, MN, USA). It is a semi-crystalline grade. The polyamide 11 (PA11 grade LMFO) was produced by Arkema (Colombes, France) under the trade name Rilsan^®^. A commercially available modified acrylic copolymer with epoxy functions (Joncryl ADR^®^-4368) was obtained from BASF (Ludwigshafen, Germany). It has an epoxy equivalent weight of 285 g/mol, an average functionality on epoxide of 9 and a weight-average molecular weight Mw of 6800 g/mol. Its chemical structure is depicted in Figure 1. Table 1 shows some properties of the used materials.

### 2.2. Blend Preparation

Before extrusion, PLA and PA11 pellets were dried under vacuum at 80 °C overnight (and Joncryl 15 min). Then, the blends were prepared in a co-rotating twin screw extruder (BC21, Clextral, Firminy, France), with a screw diameter of 25 mm and an L/D ratio of 48. A vacuum pump (Sterling Fluid Systems, Manchester, UK) was used to avoid oxidation and hydrolytic degradation during extrusion. The extrusion temperature profile was 80 °C for the feed zone, and 210 °C for all the other zones and the die. A feeding rate of 4 kg/h and a 200 rpm screw speed were applied.

Blends were prepared into two steps. First, PLA and 4 wt% Joncryl were mixed together in the twin-screw extruder, quenched in cold water and granulated. Then, modified PLA (named PLA-J4) pellets were dried under vacuum at 80 °C overnight, and secondary mixed by twin-screw extrusion with PA11 and virgin PLA. Through this way, PLA/PA11 80/20 wt/wt blends containing 0 to 3 wt% of Joncryl, based in total weight of polymer blend, (named PLA80-J0 to J3) were prepared. PLA-J4 was prepared as a masterbatch, in order to get (by dilution) high Joncryl contents in final blends named PLA80-Jx (x as Joncryl content, with x ≤ 3). Total residence time in extruder was 3 min for all blends. 

At the outlet of twin-screw extruder, blends were obtained as extruded threads, that were quenched in cold water and granulated to get pellets, used thereafter to produce samples. 

### 2.3. Standard Samples Preparation

Before samples preparation by injection molding or 3D printing, prepared blends pellets were dried under vacuum at 80 °C overnight. A part of obtained pellets were molded using a mini injection molding machine (ZAMAK Mercator, Swakina, Poland) into dog bone shaped samples (ISO 527-2:2012, 1BA standard type) for tensile test. The following parameters were used: Mold temperature = 80 °C, Barrel temperature = 210 °C, Injection pressure = 5.2 bars, Melting time of pellets before injection = 3 min.

The remaining fraction of pellets were first extruded using a single screw extruder (Yvroud, Ingre, France) at a temperature of 210 °C and a flow rate of 7 m/min, to get filaments with a diameter of 2.50 ± 0.1 mm. These filaments were then fed into a Fused Deposition Modeling (FDM) printer (A4v3, 3ntr, Oleggio, Novare, Italy) to produce samples with the same dimensions as those produced via injection molding. Printing was performed at 210 °C and a flow rate of 65 mm/s. Melted filaments were deposited with a +/− 45° angle on a plate support heated at 60 °C.

### 2.4. Morphological Characterizations

#### 2.4.1. Scanning Electron Microscopy (SEM)

A Scanning Electron Microscopy Quanta 200 FEG (FEI, Hillsboro, OR, USA) was used to observe blends morphology. The samples were fractured in liquid nitrogen, and fracture surfaces were coated with carbon. All the micrographs were recorded under high vacuum at an accelerating voltage of 3 kV.

#### 2.4.2. Selective Extraction

For each composition, 900 mg of extruded thread were immersed into 15 mL of chloroform at room temperature with stirring during 48 h to remove PLA. Then, PA11 nodules were purified by three washing/centrifugation cycles (10,000 rpm, 5 min) using chloroform and finally collected for analysis.

#### 2.4.3. Laser Diffraction Particle Size Analyzer

A Coulter LS 13230 (Beckman Coulter, Brea, CA, USA) laser diffraction particle size analyzer instrument was used to determine the size distribution of extracted PA11 nodules. Size measurements were performed using the micro liquid module (15 mL) in chloroform; obscuration was 10 ± 2%. Three measurements were performed for each sample. 

Laser diffraction particle size analyzer is an interesting alternative method to characterize dispersed phases in immiscible polymer blends. In fact, the number of dispersed phases analyzed is much larger than with conventional images analysis from electron microscopy observations (SEM or TEM (Transmission Electron Microscopy)).

### 2.5. Differential Scanning Calorimetry (DSC)

A Pyris Diamond DSC (PerkinElmer Instruments, Waltham, MA, USA) differential scanning calorimeter was used to measure the thermal characteristics of the blends. Samples of around 15 mg were cut from the pellets and put in 50 µL sealed aluminum pans. All the experiments were performed under dry nitrogen as a protective gas (20 mL/min). Three calorimetric scans were performed for each sample at a heating or cooling rate of 10 °C/min. The first heating scan, in which the thermal history was suppressed, was performed from 30 °C to 220 °C before a 2 min isothermal scan at 220 °C was applied. Then, the cooling scan went from 220 °C to 30°C, before a 2 min isothermal scan at 30 °C was applied, and finally the second heating scan was performed from 0 °C to 220 °C. 

The thermal characteristics–glass transition temperature (T_g_), cold crystallization temperature (T_cc_), enthalpy of cold crystallization (ΔH_cc_), melting temperature (T_m_) and melting enthalpy (ΔH_m_)–were determined from the second heating scan. The degree of crystallinity (X_C_) of the PLA and PA11 into the blend were calculated using the following equation:

(1) $$X_{c}=\frac{△H_{m}-△H_{\mathrm{cc}}}{△{H_{m}}^{0}\times w}\times 100,$$

ΔH_m_^0^ is the enthalpy of fusion per mole of repeating unit of perfect crystal of infinite size (totally crystalline polymer). We considered ΔH_m_^0^ (PLA) = 93 J/g [29] and ΔH_m_^0^ (PA11) = 200 J/g [30]). w is the weight fraction of polymer into the blend. 

At least two measurements were performed for each sample. 

### 2.6. Rheological Tests

All samples were prepared using a mini injection molding machine (ZAMAK Mercator, Swakina, Poland) at 210 °C for 3 min with a constant pressure of 5.2 bars in the form of disks of 25 mm diameter and 1.5 mm thickness. All samples were dried in a vacuum oven at 80 °C for 24 h before the rheological tests.

The rheological analysis was carried out using a controlled-stress rheometer (MCR 702 TwinDrive, Anton Paar, Graz, Austria) in a parallel plate geometry (25 mm diameter) and a 1.3 mm gap at 210 °C under dry nitrogen flow. The linear viscoelastic region was determined using strain sweep tests for the neat polymers at 6.28 rad/s frequency. According to the obtained results, the frequency sweep tests were performed at a strain of 2%, from 100 to 0.01 rad/s. The stability of samples under the test conditions was checked using a time sweep test and a less than 7% drop in the complex viscosity was observed in the experimental time scale of 60 min.

### 2.7. Tensile Tests

Tensile properties were obtained using a universal tensile machine Zwick Z010 (Zwick Roell, Ulm, Germany). For each composition, at least five dog bone shaped samples (ISO 527-2:2012 type 1BA standard), prepared by injection molding or FDM as described previously, were tested in ambient conditions (around 23 °C and 50% relative humidity).

Tensile tests were performed first at a cross-head speed of 5 mm/min to determine Young modulus (E), then at 100 mm/min until material failed. Maximum stress (σ_m_), elongation at break (ε_b_) and stress at break (σ_b_) were recorded.

### 2.8. Porosity Measurement

Porosity, defined by the ratio of voids volume (V_voids_) to total volume (V_total_), of FDM printed samples was determined to judge their intrinsic brittleness with the following equation:

(2) $$\mathrm{Porosity}(\%)=\frac{V_{\mathrm{voids}}}{V_{\mathrm{total}}}=\left[1-\frac{d_{\mathrm{apparent}}}{d_{\mathrm{absolute}}}\right]\times 100,$$

where d refers to density, apparent or absolute. 

Apparent density is defined as the ratio between the global mass and the global volume of a material, as it appears including pores. Global mass was measured by weighing samples (complete dog bone shaped samples) and volume was determined measuring exact dimensions samples with a vernier caliper. 

Absolute density refers to the real density of a material, which means the ratio between the global mass and the solid volume of a material excluding pores. This density was determined using an helium pycnometer (Accu Py 1330, Micromeritics, Norcross, GA, USA).

Two samples by composition were used for these measurements. 

## 3. Results and Discussion 

### 3.1. Effects of Joncryl on Blends Morphology and Properties

#### 3.1.1. Blends Morphology

**Figure 2:**
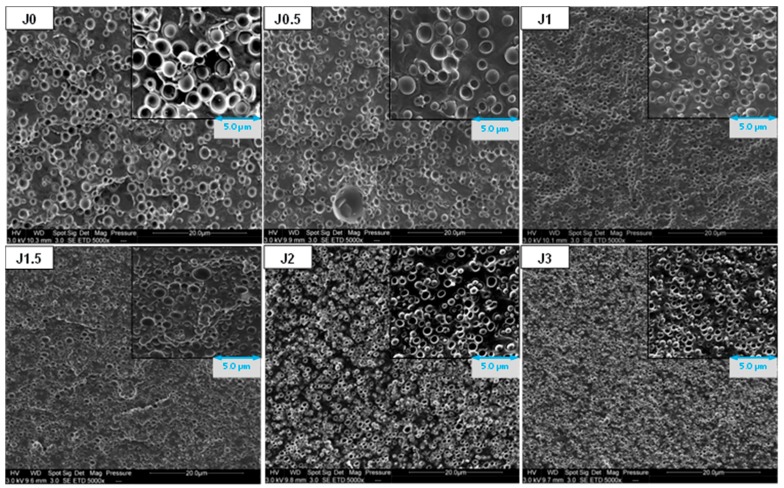
SEM observations of extruded samples threads in transversal direction.

Figure 2 shows SEM images of PLA/PA11 blends prepared by melt extrusion. As expected, all blends exhibit sea island morphology, with a PLA matrix and PA11 dispersed droplets. We can note that PA11 dispersed phases diameter seems to decrease with increasing Joncryl content. For Joncryl content higher than 1.0 wt%, no significant evolution could be observed. 

**Figure 3:**
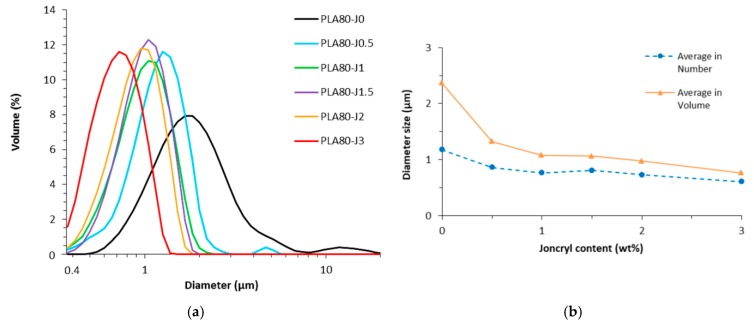
Size distribution curves (**a**) and emulsification curve (**b**) of PA11 dispersed phases diameter as a function of Joncryl content.

To confirm this trend, size distribution and average diameter of extracted PA11 nodules, after PLA dissolution by chloroform, were determined using laser diffraction analysis. As we can see on Figure 3, a reduction of PA11 dispersed phases volume diameter with increasing Joncryl content is well observed. Joncryl addition leads to submicronic nodules (Table S1). Compared to neat blend, we can also note a narrowing of the size distribution with Joncryl addition, which is in good correlation with SEM observations. 

The observed PA11 diameter reduction shows that Joncryl has an emulsifying effect on the blend, because of coalescence suppression. Such phenomenon could be associated with compatibilization due to the chemical reactivity of the epoxide function of Joncryl with the polymers of the blend, i.e., amine and acid chain end of PA11 and PLA, respectively, as concluded by Walha et al. [21]. Moreover, regarding the emulsification curve, a saturation of the reaction at the interface occurs around 1.0 wt% of Joncryl.

#### 3.1.2. Thermal Properties and Crystallinity 

**Figure 4:**
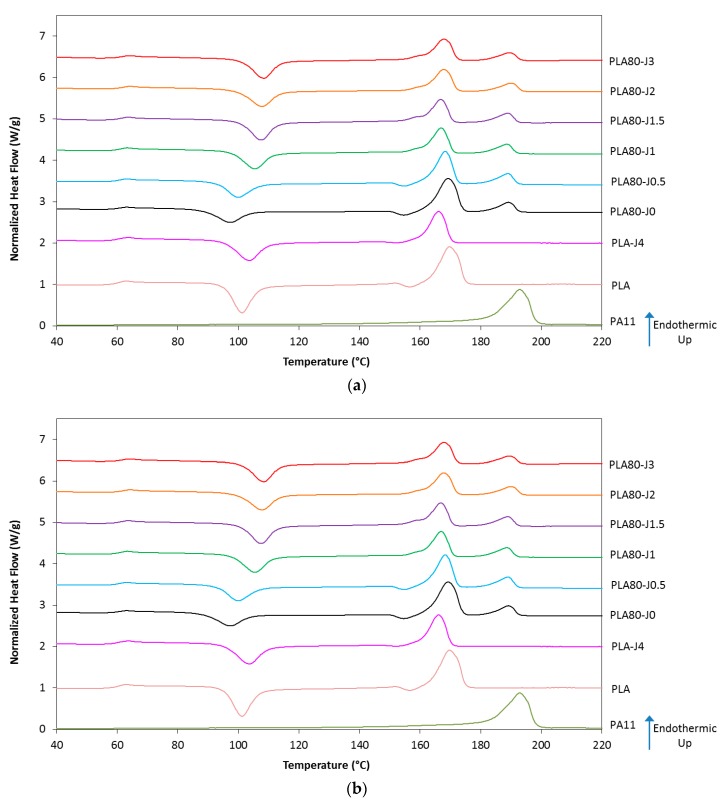
DSC thermograms of the second heating of neat polymers (**a**) and PLA80-Jx blends (**b**).

DSC thermograms and data collected from the second heating scan of neat polymers (PLA, PLA-J4 and PA11) and their blends PLA80-Jx with various Joncryl content (x) are presented in Figure 4 and Table 2.

The addition of PA11 to the PLA (PLA80-J0) dramatically increases the PLA crystallinity degree (X_c_). PA11 domains might act as nucleating centers and thereby enhance the crystallization of the PLA in the blend [31]. For compatibilized blends, the higher the Joncryl content, the higher the PLA cold crystallization temperature T_cc_ in the blend. Thus, T_cc_ is shifted from 98 °C (neat blend) to 109 °C (PLA80-J3), which represents an increase of 11 °C. At the same time, a slight increase of ΔH_cc_ is observed. For Joncryl content higher than 0.5 wt%, we can also note the disappearance of the second PLA cold crystallization peak. Finally, PLA crystallinity degree drops with increasing Joncryl content from 22.4% to 1.6%. Similar changes can be noted if PLA-J4 data are compared with pure PLA data. 

It shows that the observed crystallization modifications are related to structural modification of PLA chains induced by Joncryl. As a chain extender, Joncryl leads to increase length chains and their molecular weight. Hence, long-chain branched structures are formed in Joncryl-modified PLA that increase molecular weight and decrease chains mobility [32]. The presence of branches disrupts the packing of polymer chains, thus preventing crystallization happening during the cooling step. As a consequence, crystallinity degree decreases. The reduced chain mobility is responsible for the increased cold crystallization temperature, as observed by Najafi et al. [33]. 

Hence, it can be considered that the same behavior also occurs in blends, its intensity being governed by the PLA-J4 amount. No significant change concerning glass transition and melting temperatures of each polymer into blends was observed.

#### 3.1.3. Rheological Behavior

**Figure 5:**
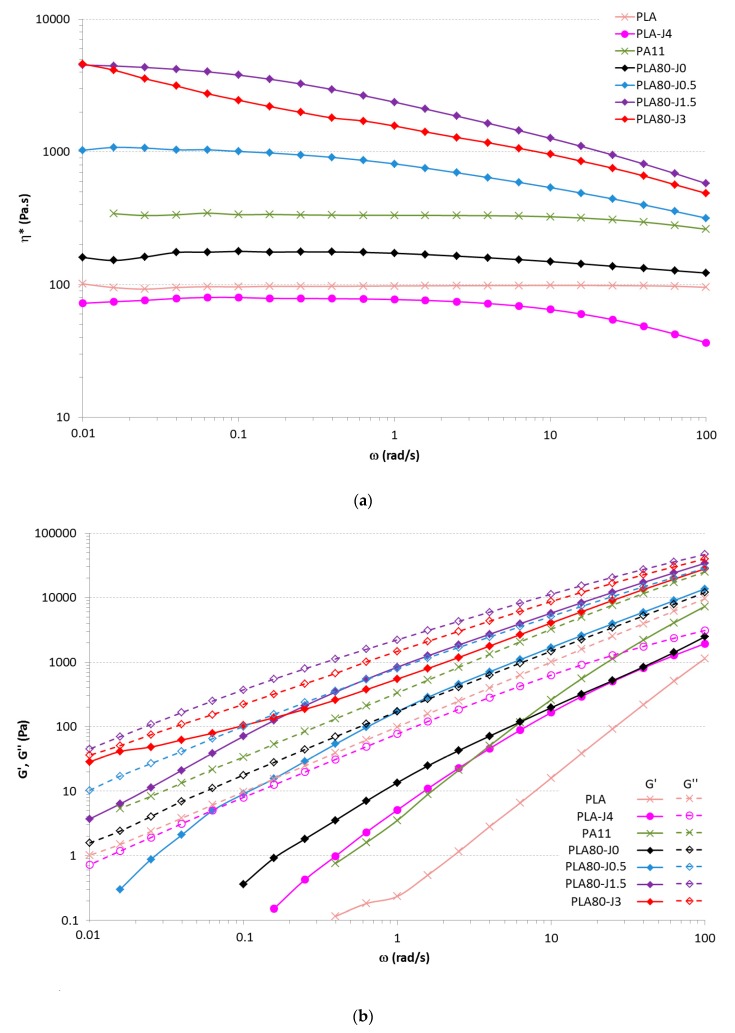
Complex viscosity (**a**), storage modulus (full lines) and loss modulus (dashed lines) (**b**) versus the angular frequency for PLA, PLA-J4 and PA11 PLA80-Jx blends (x = 0; 0.5; 1.5; 3).

Figure 5 illustrates the evolution of the complex viscosity η*, the storage modulus G’ and the loss modulus G” versus angular frequency of PLA, PLA-J4, PA11 and PLA80-Jx blends (for x = 0; 0.5; 1.5; 3). First, we can note that PLA-J4 has a lower complex viscosity than PLA, but with a crossover point (between G’ and G’’) occurring at lower frequency than PLA. These results might be explained by the fact that some PLA chains have reacted with Joncryl, increasing the molecular weight or chain branching and then increasing the relaxation time of the chain [34]. Indeed, Wang et al. [35] have observed a slight crosslinking and an increase in molecular weight of PLA in the presence of Joncryl. However, in the other hand, there is probably free Joncryl that has not reacted with any PLA chains, because it was introduced in excess, and, as Joncryl is a small molecule (η_0_ = 30.6 Pa·s), it dramatically contributes to decrease the viscosity of the whole PLA-J4 sample.

The complex viscosity curve of the neat PLA/PA11 blend is located between the values obtained for the neat polymers. When PLA-J4 is added, complex viscosity is increased with increasing Joncryl content in the blend over the whole frequency range. This result is in accordance with Walha et al. works [21]. These authors showed that such behavior can be associated with chemical reaction taking place between PLA and PA11 and indicates an increase of the intermolecular interactions of the blend system due to this interfacial reaction. A narrowing of the Newtonian plateau at low frequency is also observed. It must be noticed that, at low frequency, PLA80-J3 exhibit a yield behavior compared to the other PLA80-Jx formulations. This yield behavior is identified on the shape of η* at low frequency that does not reach a plateau and on the behavior of G’ which tends to reach a plateau value at low frequency. These behaviors are often reported in reactive compatibilization [36,37,38]. 

Moreover, PLA80-J3complex viscosity is slightly lower than that of PLA80-J1.5. We can assume that, between 1.5 and 3 wt% Joncryl, both amine and acid chain end of PA11 and PLA have completely reacted. The saturation is attained and the excess of Joncryl that didn’t react contributes to decrease the complex viscosity. In the same way as PLA-J4, an excess of Joncryl decreases the complex viscosity of the blend PLA80-J3. 

G’ of the neat blend is in between that of the pure polymers from 100 to 10 rad/s, then it diverges from the rule of mixture as G’ of the PLA80 blend become higher than G’ of the PA11 at lower frequency. This is due to the well-known shape relaxation behavior [39]. G’ of PLA80-Jx samples raises with increasing Joncryl content, because of the appearance of some extended/branched chains as well as an increase of the entanglement density.

### 3.2. Comparison Between Injected and FDM Samples Morphology and Mechanical Properties

#### 3.2.1. FDM Filaments and Dog Bone Shaped Samples Morphology

**Figure 6:**
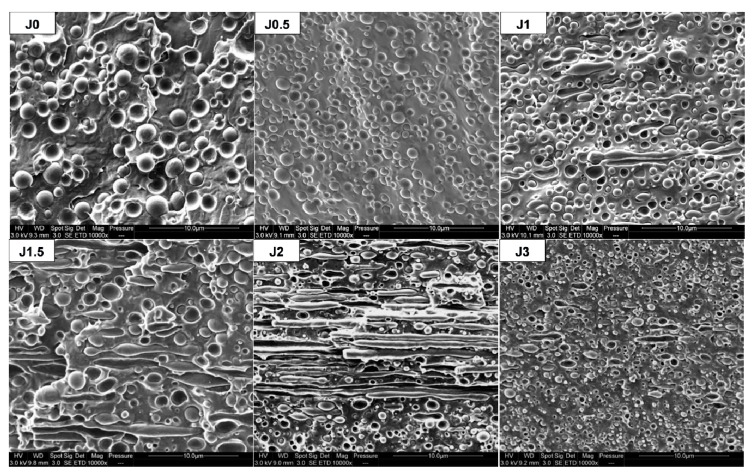
SEM observations of FDM filaments in longitudinal direction.

SEM observations of cross-sections of FDM filaments prepared by single-screw Yvroud extrusion are shown in Figure 6. They can be compared with Figure S1, depicting SEM micrographs of cross-sections corresponding to the extrusion direction (longitudinal) performed on extruded threads obtained by twin-screw extrusion. 

For both extruded threads and FDM filaments, a reduction of PA11 dispersed phase diameters with increasing Joncryl content is observed, similarly to the previous observations about transverse cross-sections (Figure 2). Elongated PA11 nodules are observed for a Joncryl content between 1 and 3 wt% in FDM filaments. Moreover, we can note that the higher the Joncryl content, the more important the number and length of elongated phases, except for PLA80-J3. 

It can be considered that the presence of elongated phases results from a decrease of the interfacial tension, due to the compatibilizing role of the agent Joncryl. But, it could also be the result of a change in viscosity ratio, that affects the droplets coalescence and breakup mechanisms which governs the morphology of the polymer blend [40,41]. Indeed, if the matrix viscosity decreases, the viscosity ratio, defined as the ratio between dispersed phase viscosity and the matrix one’s, increases. In our case, it was shown previously that from 1.5 wt% of Joncryl in the blend, the viscosity starts to decrease. Hence from 1.5 wt% of Joncryl, the polymer matrix is composed of PLA and unreacted Joncryl, and the blend viscosity is decreased. This promotes PA11 elongation by impeding thread break-up.

Concerning PLA80-J2 blend, the aspect ratio of PA11 phases is more important for FDM filaments than for extruded threads. Supplementary elongation in FDM filament could be explained by the higher mechanical stresses applied during single-screw extrusion, compared to the ones applied during twin-screw extrusion to get extruded threads.

**Figure 7:**
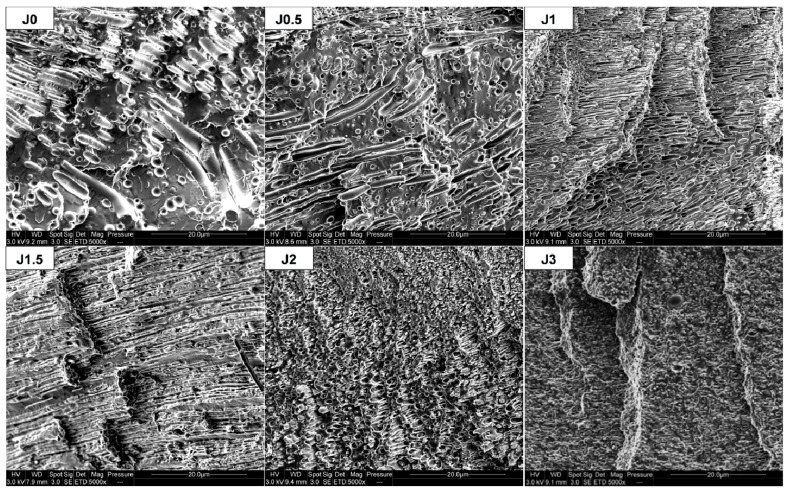
SEM observations of FDM dog bone shaped samples in longitudinal direction.

For dog bone shaped samples, processed through 3D printing, elongated PA11 dispersed phases also appear for all compositions (Figure 7). Figure 7 shows that FDM process induces supplementary elongation, in addition to filament extrusion.

#### 3.2.2. Mechanical Properties

**Figure 8:**
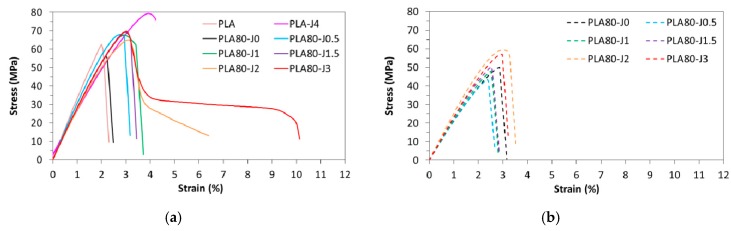
Stress–strain curves of injected PLA, PLA-J4 and PLA80-Jx blends (**a**) and FDM printed PLA80-Jx blends (**b**).

The results of tensile properties after injection show that the brittle properties of PLA can be modified by the addition of Joncryl. The elongation at break is doubled, since it increases from 2.1% (pure PLA) to 4.3% (PLA-J4), and maximal stress increases too. But, in contrast, adding ductile PA11 to brittle PLA does not improve significantly its mechanical properties, since PLA80-J0 blend shows a similar behavior than neat PLA (Figure 8a). That is certainly due to a lack of adhesion between polymers, because of their immiscibility and incompatibility.

Blending PLA-J4 with PLA and PA11 entails an increase on elongation at break (after injection), which tends to increase with increasing Joncryl content. At high Joncryl content 2 and 3 wt%, a ductile behavior is observed, with the highest elongation at break of 6.9% and 9.8% respectively (Figure 8a, Table S2). No significant change of tensile modulus and maximal stress is noticed compared to the neat blend. Such behavior indicates that an improved interface between PLA and PA11 was established by reactive compatibilization with Joncryl. To confirm this conclusion, SEM observations of tensile fracture surface for injected samples were carried out.

**Figure 9:**
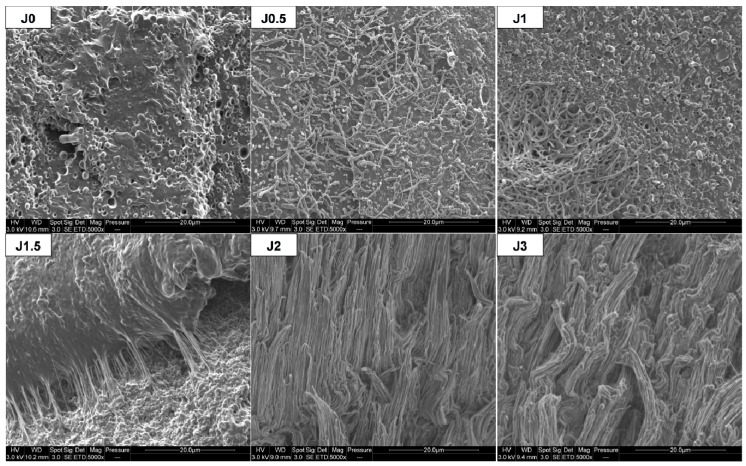
SEM observations of tensile fracture surface for injected PLA80-Jx.

In comparison with the neat blend, elongation of PA11 dispersed phases in the compatibilized blends was observed. After tensile test, this phenomenon is all the more important since Joncryl content is high and two cases can be distinguished (Figure 9):

- For 0≤ x ≤ 1.5: PA11 dispersed phases extend individually but not PLA matrix, because of its intrinsic brittle behavior and owing to the lack of interfacial adhesion, leading to a clear brittle failure.
- For x = 2 or 3: PA11 dispersed phases extend with PLA matrix and form an elongated structure, leading to a ductile failure.

Hence, thanks to a sufficient interfacial adhesion, PA11 dispersed phases could transfer mechanical stress, more easily at high Joncryl content, to enable a more ductile behavior.

PA11 dispersed phases size reduction could also contribute to increase elongation at break. Indeed, this phenomenon leads a better homogeneity of PA11 nodules into blend, as evidenced by SEM micrographs (Figure 2) and the narrowing of the size distribution (Figure 3), beneficial to tensile properties enhancement. 

Concerning the samples prepared by FDM, as we can observe in Figure 8b, FDM printed samples show a brittle mechanical behavior, including PLA80-J2 and PLA80-J3 blends that exhibited a ductile behavior when processed by injection molding. As observed for injected samples, maximal stress tends to increase with increasing Joncryl content, to reach a maximum of 58.8 MPa for PLA80-J2 (Table S3). In comparison to the neat blend, Young modulus values are quite similar, except for PLA80-J2 and PLA80-J3 exhibiting a modulus higher than 3000 MPa. 

**Figure 10:**
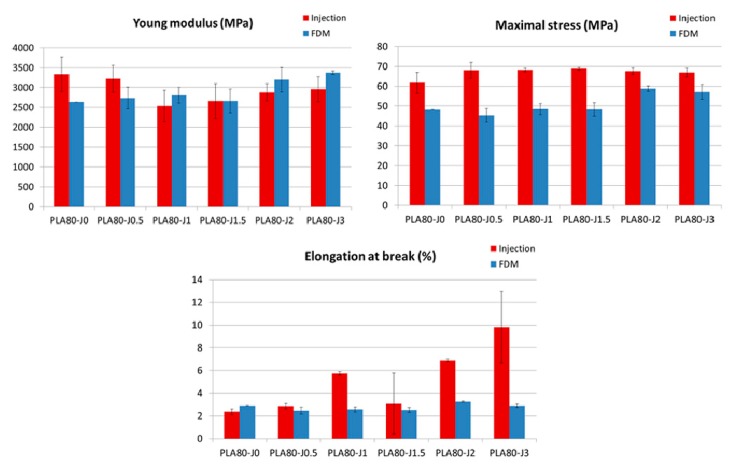
Injected versus FDM printed samples mechanical properties.

If we compare FDM printed samples tensile properties to the ones of injected samples, FDM process can allow more rigid samples to be obtained, that means higher Young’s moduli, especially at high Joncryl content (Figure 10). This can be ascribed to the aspect ratio of PA11 elongated dispersed phases, more important for printed samples in comparison to this for injected dog bone shaped samples (Figure 7 and Figure S2). 

Nevertheless, it can be mainly noticed that FDM printed samples are more brittle compared to injected ones, since maximal stress and elongation at break are lower. 

Two parameters related to FDM process can be pointed out to explain this difference: the lack of adhesion between deposited filaments and the porosity. FDM consists in the deposit of melted polymer flow on a support as aligned threads, that requires enough adhesion between deposited threads in a same layer or between the subsequent layers. 

In the FDM process, melted filaments are extruded through a circular nozzle and deposited threads are circular, leading to trapped air between them, even if a partial merging and compression ensure cohesion between them. As a result, voids appear and represent potential cracking areas under mechanical stress. 

Both parameters are involved in the cavitation process, leading to the material’s failure. To determine to which extent these parameters affect tensile properties of printed samples, their morphology after the tensile test was observed and their porosity was determined. 

#### 3.2.3. Printed Samples Morphology and Porosity 

**Figure 11:**
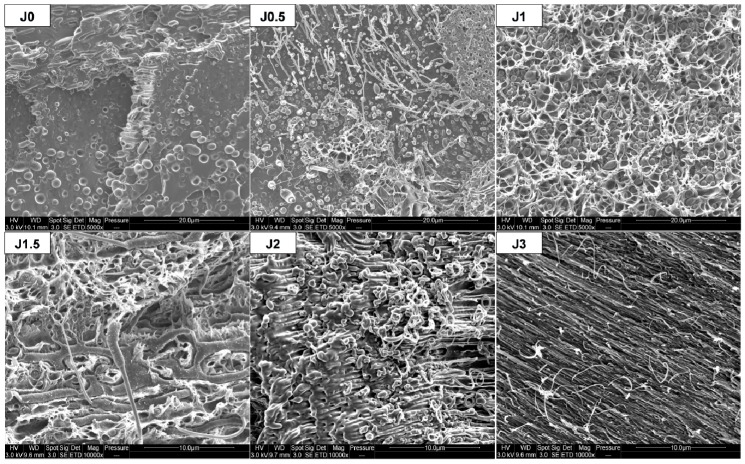
SEM observations of tensile fracture surface for printed samples.

After the tensile test, fracture surface morphology of FDM printed dog bone shaped samples varies as a function of Joncryl content, as observed for injected samples (Figure 9). Elongation of PA11 dispersed phases was observed, all the more important since Joncryl content is high (Figure 11). Such observations suggest that adhesion between polymers in the blend would be high enough to ensure a good mechanical transfer leading to enhanced mechanical properties. 

Nevertheless, the experimental results do not support these assumptions. Hence, the lack of adhesion between the deposited threads and the existence of porosity between them seem to govern the mechanical behavior and can account for the brittleness of the printed samples in comparison with the injected ones.

**Figure 12:**
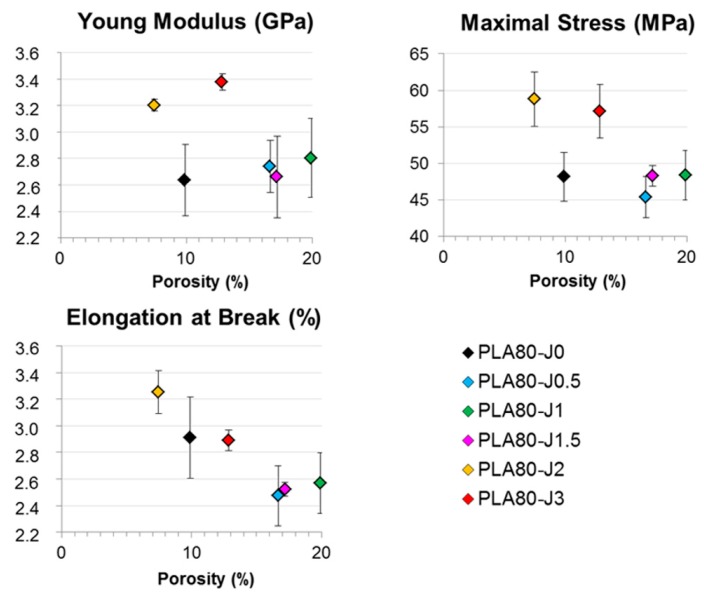
Mechanical properties as a function of porosity for FDM printed samples.

Table 3 summarizes values obtained for porosity and absolute density of FDM printed samples. It can be noticed that samples have a similar absolute density and their porosity is quite important, higher to 10% on the whole. The highest porosities were measured for specimens with Joncryl content between 0.5% and 1.5%. As shown Figure 12, there is a good correlation between porosity and mechanical properties. Indeed, these compositions have the poorest mechanical properties compared to other samples. Several hypotheses can be proposed to explain why porosity is higher for these samples compared to the other compositions.

First, viscosity of melted polymer during the FDM process could affect the deposition features of extruded thread. If the melted polymer is too viscous, the extruded thread will undergo few deformations and is unable to merge easily with the adjacent deposited thread, which creates voids. On the contrary, if melted polymer is less viscous, less air will be trapped, because the extruded thread will spread and could merge easily with the adjacent deposited thread.

Another parameter that can explain porosity development is the crystallization process and the crystallization rate of melted deposited polymer. If polymer has a fast crystallization and a high crystallinity after cooling, it will entail a shrinking of the deposited thread, which couldn’t easily merge with the adjacent deposited thread and will create pores.

Further investigations would be necessary to determine the influence of these parameters. 

## 4. Conclusions

Incorporation of Joncryl, a multifunctionalized epoxide, into a PLA/PA11 80/20 blend greatly improved properties of such a polymer blend, depending on its content. Studies of the morphological, thermal, rheological and mechanical properties of blends revealed that Joncryl acted as a compatibilizer. A reduction in PA11 dispersed phases diameter was observed, indicating compatibilization through coalescence suppression. Regarding the emulsification curve, saturation occured at 1.0 wt% of Joncryl.

PLA matrix cold crystallization changes in the compatibilized blends highlighted extension and branching reactions on PLA chains modified by Joncryl. The extent of such reactions is enhanced with increasing Joncryl content. Rheological and mechanical properties followed the same trend.

Complex viscosity of blends during frequency sweep tests increased from 0 to 1.5 wt% of Joncryl. Such an increase, mostly pronounced at low frequency, is assigned to an interfacial reaction between PLA and PA11 with Joncryl, as coupling agent. At 3 wt% of Joncryl, a slight reduction of complex viscosity was observed, that revealed the presence of an excess of Joncryl in the PLA matrix that didn’t react. Tensile tests showed an enhancement of ultimate mechanical properties (maximal stress and elongation at break), especially for blends containing 2 and 3 wt% of Joncryl exhibiting a ductile behavior. This results from an improved interfacial adhesion through reactive compatibilization with Joncryl, as proved by SEM observations of tensile fracture surfaces. As a matter of fact, tensile tests show an elongation of PA11 ductile dispersed phases. Moreover, at high Joncryl content, the PLA matrix could be stretched with PA11 dispersed phases thanks to a high enough interfacial adhesion.

Preparation of compatibilized blends by FDM process led to a significant elongation of PA11 dispersed phases in FDM filaments and dog bone shaped specimens. Occurrence of such morphology was observed for Joncryl content from 1 to 2 wt%. The aspect ratio of PA11 dispersed phases increases with Joncryl content. Despite this specific morphology, a priori beneficial to better tensile properties, FDM printed samples were more brittle compared to injected ones. Their maximal stress and elongation at break were lower. It is nevertheless interesting to note that higher Young’s moduli could be obtained with FDM samples at 2 and 3 wt% of Joncryl, probably as a result of highly elongated PA11 dispersed phases. FDM printed samples’ brittleness can be explained mainly by a lack of adhesion and porosity between deposited extruded threads during FDM process. 

## Figures and Tables

**Table 1:** Characteristics of the raw materials.

Material	Density (g/cm^3^)	T_g_ (°C)	T_cc_ (°C)	T_m_ (°C)
PLA	1.24	59 *	101 *	170 *
PA11	1.02	-	-	190 *
Joncryl	1.08	54	-	-

* From Differential Scanning Calorimetry (DSC) experimental data.

**Table 2:** DSC results obtained from the second heating scan.

Samples	T_g_ PLA (°C)	T_cc_ PLA (°C)	ΔH_cc_ PLA (J/g)	T_cc2_ PLA (°C)	ΔH_cc2_ PLA (J/g)	T_m_ PLA (°C)	ΔH_m_ PLA (J/g)	T_m_ PA11 (°C)	ΔH_m_ PA11 (J/g)	X_c_ PLA (%)	X_c_ PA11 (%)
PLA	59	101	32.7	156	1.6	170	40.4	-	-	6.9	-
PLA-J4	59	104	26.3	152	0.4	166	30.6	-	-	4.3	-
PA11	-	-	-	-	-	-	-	193	49.2	-	24.6
PLA80-J0	60	98	18.0	155	2.6	170	34.7	190	8.9	22.4	22.2
PLA80-J0.5	60	101	21.7	155	1.6	169	29.9	190	9.4	11.0	23.6
PLA80-J1	60	106	21.7	-	-	168	24.4	189	9.1	3.6	23.0
PLA80-J1.5	60	107	22.4	-	-	167	23.5	189	9.4	1.6	23.8
PLA80-J2	61	108	21.7	-	-	168	23.6	190	8.9	2.6	22.7
PLA80-J3	60	109	22.8	-	-	168	23.9	189	8.2	1.6	21.2

**Table 3:** Values obtained for porosity and absolute density of FDM printed samples.

Samples	Porosity (%)			Absolute Density (g/cm^3^)		
PLA80-J0	9.91	±	0.29	1.1981	±	0.0007
PLA80-J0.5	16.66	±	2.61	1.1986	±	0.0033
PLA80-J1	19.93	±	0.60	1.2006	±	0.0015
PLA80-J1.5	17.19	±	6.48	1.2026	±	0.0003
PLA80-J2	7.47	±	4.90	1.1974	±	0.0007
PLA80-J3	12.86	±	0.37	1.1945	±	0.0057

## Supplementary Materials

The following are available online at http://www.mdpi.com/1996-1944/12/3/485/s1, Figure S1: SEM observations of twin-screw extruded threads samples in longitudinal direction, Figure S2: SEM observations MEB of injected dog bone shaped samples, Table S1: Diameters of PA11 dispersed phases extracted from PLA80-Jx blends, Table S2: Mechanical properties of injected neat polymers and PLA80-Jx blends, Table S3: Mechanical properties of FDM printed PLA80-Jx blends.
