# Supplementary material for: Reactive Compatibilization of PLA/PA11 Blends and Their Application in Additive Manufacturing
Source: Materials (Basel). 2019 Feb 5;12(3):485. doi: 10.3390/ma12030485 (PMC6384960; doi:10.3390/ma12030485)
Supplement: Supplementary file 1 [file materials-12-00485-s001.pdf]

materials

Supplementary Materials

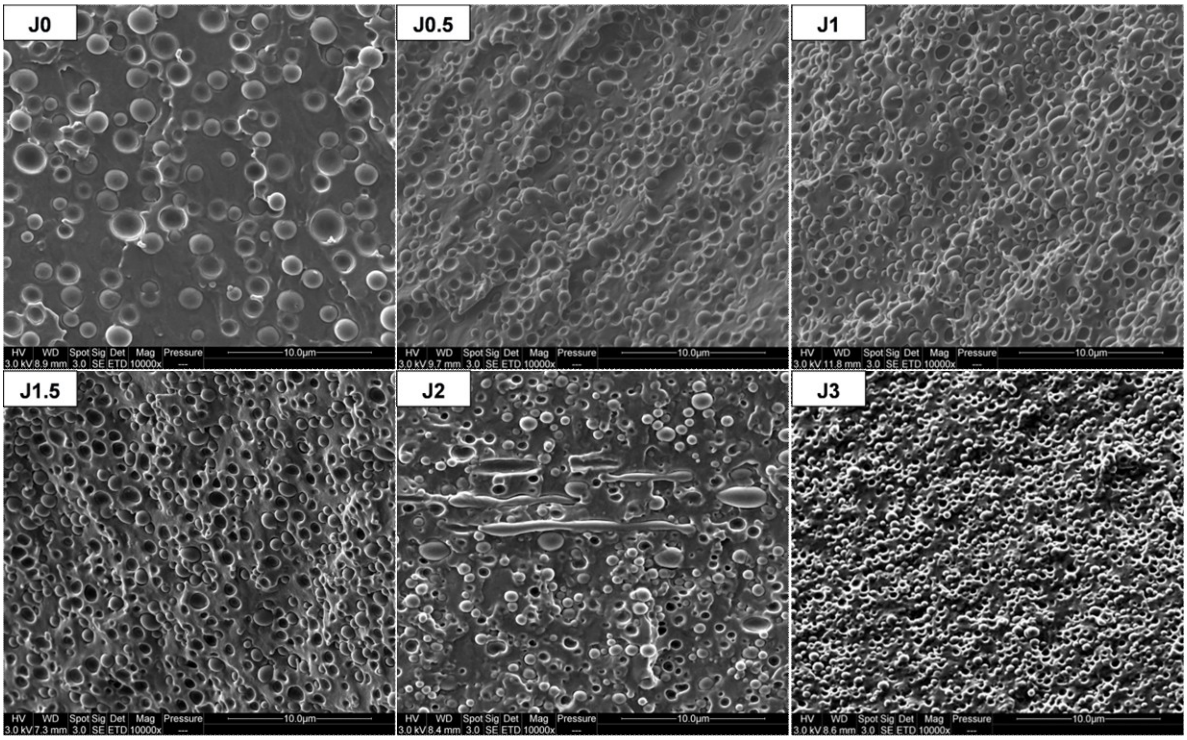

Figure S1. SEM observations of twin-screw extruded threads samples in longitudinal direction

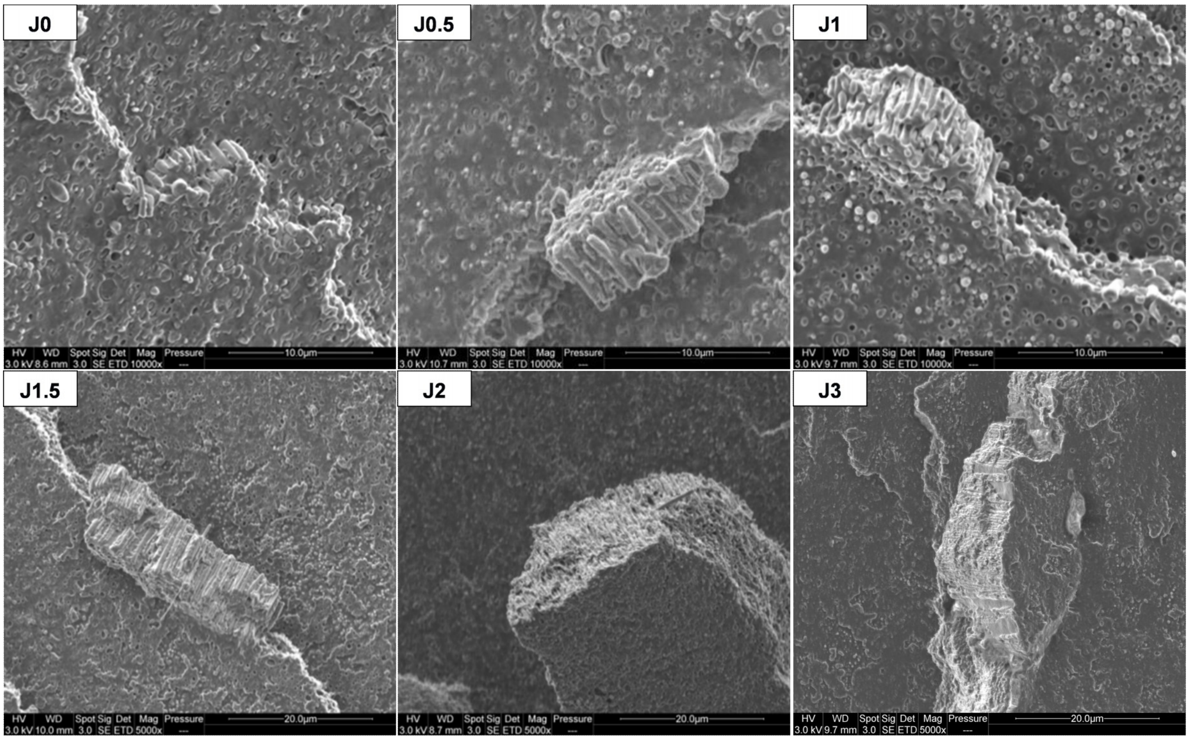

Figure S2. SEM observations of injected dog bone shaped samples

Table S1. Diameters of PA11 dispersed phases extracted from PLA80-Jx blends

	**Median volume diameter** d_{v50} **(μm)**	**Average volume diameter** d_v **(μm)**	**Average number diameter** d_n* **(μm)**
PLA80-J0	1.86	2.37	1.18
PLA80-J0.5	1.27	1.32	0.86
PLA80-J1	1.05	1.08	0.77
PLA80-J1.5	1.05	1.06	0.81
PLA80-J2	0.96	0.98	0.73
PLA80-J3	0.74	0.76	0.60

* Calculated considering dispersed phases volume as the one of a sphere

Table S2. Mechanical properties of injected neat polymers and PLA80-Jx blends

	**E (MPa)**			**σ_m (MPa)**			**σ_b (MPa)**			**ε_b (%)**		
PLA	3817	±	436	65.7	±	5.3	65.7	±	5.3	2.1	±	0.3
PLA-J4	3329	±	653	79.1	±	0.9	75.7	±	1.4	4.3	±	0.3
PA11	1499	±	110	46.5	±	1.5	28.9	±	0.8	24.5	±	3.9
PLA80-J0	3333	±	342	61.7	±	4.0	61.7	±	4.0	2.4	±	0.2
PLA80-J0.5	3220	±	397	68.0	±	0.9	66.4	±	1.5	2.9	±	0.1
PLA80-J1	2535	±	442	68.2	±	0.7	40.8	±	24.3	5.7	±	2.7
PLA80-J1.5	2658	±	213	68.8	±	1.7	67.0	±	1.9	3.1	±	0.1
PLA80-J2	2882	±	318	67.6	±	2.2	33.0	±	23.1	6.9	±	3.1
PLA80-J3	2959	±	204	66.8	±	2.7	20.6	±	0.8	9.8	±	1.5

Table S3. Mechanical properties of injected neat polymers and PLA80-Jx blends

	**E (MPa)**			**σ_m (MPa)**			**σ_b (MPa)**			**ε_b (%)**		
PLA80-J0	2636	±	272	48.2	±	3.3	48.2	±	3.3	2.9	±	0.3
PLA80-J0.5	2738	±	197	45.4	±	2.8	45.4	±	2.8	2.5	±	0.2
PLA80-J1	2804	±	299	48.4	±	3.4	48.4	±	3.4	2.6	±	0.2
PLA80-J1.5	2661	±	307	48.3	±	1.4	48.3	±	1.4	2.5	±	0.1
PLA80-J2	3201	±	44	58.8	±	3.7	57.8	±	3.1	3.3	±	0.2
PLA80-J3	3376	±	64	57.1	±	1.2	57.1	±	1.2	2.9	±	0.1
